# MAF-Net: A multimodal data fusion approach for human action recognition

**DOI:** 10.1371/journal.pone.0319656

**Published:** 2025-04-09

**Authors:** Dongwei Xie, Xiaodan Zhang, Xiang Gao, Hu Zhao, Dongyang Du

**Affiliations:** 1 Guangdong Engineering Polytechnic, Guangzhou, China; 2 Guangdong Eco-Engineering Polytechnic, Guangzhou, China; 3 Guangdong Justice Police Vocational College, Guangzhou, China; 4 Zhongkai University of Agriculture and Engineering, Guangzhou, China; Purdue University, UNITED STATES OF AMERICA

## Abstract

3D skeleton-based human activity recognition has gained significant attention due to its robustness against variations in background, lighting, and viewpoints. However, challenges remain in effectively capturing spatiotemporal dynamics and integrating complementary information from multiple data modalities, such as RGB video and skeletal data. To address these challenges, we propose a multimodal fusion framework that leverages optical flow-based key frame extraction, data augmentation techniques, and an innovative fusion of skeletal and RGB streams using self-attention and skeletal attention modules. The model employs a late fusion strategy to combine skeletal and RGB features, allowing for more effective capture of spatial and temporal dependencies. Extensive experiments on benchmark datasets, including NTU RGB+D, SYSU, and UTD-MHAD, demonstrate that our method outperforms existing models. This work not only enhances action recognition accuracy but also provides a robust foundation for future multimodal integration and real-time applications in diverse fields such as surveillance and healthcare.

## Introduction

With the rapid development of Internet of Things (IoT) technology and the increasing demand for Human-Computer Interaction (HCI), action recognition technology plays a crucial role in these fields. Action recognition can be applied in various scenarios, such as surveillance, healthcare, and smart homes, and is also widely used in emerging fields like Augmented Reality (AR) and Virtual Reality (VR) [1–4]. In these applications, automated and accurate action recognition systems can significantly enhance the intelligence level of devices and improve user experience. Consequently, the efficient and accurate recognition and analysis of human actions has become a research hotspot in recent years [5–8]. Particularly, with the breakthroughs in deep learning technology, action recognition methods based on Deep Neural Networks (DNN) have made remarkable progress. Against this backdrop, researchers have begun to explore the combination of multimodal data sources, with the integration of RGB video modality and skeleton sequence modality showing great potential [9–13]. This combination not only improves the accuracy of action recognition but also achieves better robustness and generalization in complex scenarios.

In action recognition, RGB video and skeleton sequence modalities provide complementary information that enhances the accuracy of human motion analysis [14–16]. RGB video captures rich spatial information, including the trajectory of human movements and interactions with objects in the environment. For instance, in a grasping action, the RGB modality can reveal the motion trajectory of the hand, the shape of the object, and the relative positioning between the hand and the object, offering crucial contextual cues for action understanding. In contrast, the skeleton sequence modality represents human poses and limb movements through keypoints, primarily focusing on temporal motion patterns [17,18]. It excels at tracking the trajectory of body parts but lacks the spatial context needed to accurately interpret human-object interactions. Therefore, while RGB video provides detailed visual information, the skeleton sequence emphasizes the geometric structure of human motion, making these two modalities highly complementary for action recognition tasks.

Existing action recognition methods that fuse RGB video and skeleton sequence modalities face several notable challenges. Firstly, processing RGB video typically relies on computationally intensive 3D Convolutional Neural Networks (3D CNNs) or hierarchical 2D Convolutional Neural Networks (2D CNNs) [19–22]. Although these methods effectively capture spatiotemporal information within video sequences, their high computational cost makes them less suitable for resource-constrained or real-time applications. Furthermore, the efficiency of multimodal information fusion has become a bottleneck, with many approaches increasing model complexity and inference time as they aim to improve recognition accuracy, thus limiting the feasibility of real-world applications [23–26]. To address these issues, some approaches have adopted the strategy of extracting single-frame RGB images from videos, which are processed by 2D convolutional networks to reduce computational overhead. RGB frames can provide robust spatiotemporal information, particularly in scenarios where human-object interactions (such as with bottles or caps) persist throughout the video, making it effective to extract spatial information from intermediate frames [27–29]. Compared to full RGB video processing, using 2D convolution-based approaches significantly reduces network complexity while retaining most of the critical information.

**Fig 1:**
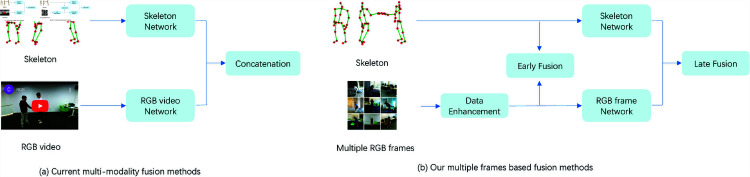
Fusion methods.

Replacing RGB video with individual frames can effectively reduce computational complexity, but inevitably leads to a loss of temporal information from the video stream. This information loss hinders the ability to share consistent feature representations between the RGB and skeleton streams, ultimately impacting recognition accuracy. To address this issue, this paper proposes an innovative network architecture called MAF-Net, which aims to optimize the fusion strategy between RGB video and skeleton sequences, reducing computational complexity while enhancing the efficiency and accuracy of action recognition. Compared to traditional cascade or weighted-sum fusion methods [65, 70] (as shown in Fig 1a), this method introduces data augmentation for RGB frame images and early cross-modal feature fusion (as shown in Fig 1b). In the early fusion stage, the method generates an attention mask from the projection of the skeleton sequence onto the RGB frames, guiding the RGB network to focus on high-information regions related to limb movement, thereby improving the effectiveness of feature extraction. Additionally, a self-attention mechanism is integrated into the RGB frame network to suppress background noise and further enhance the extraction of relevant information.In the final fusion phase, a cross-modal attention integration module is utilized to seamlessly integrate skeletal data with RGB data, enabling comprehensive modal fusion and enhancing the overall action recognition performance. This two-stage fusion strategy significantly enhances the sharing of multimodal features and recognition accuracy while maintaining manageable model complexity.

To validate the effectiveness of MAF-Net, experiments were conducted on three public datasets: NTU RGB+D, SYSU, and UTD-MHAD. The experimental results demonstrate that MAF-Net significantly outperforms existing multimodal action recognition methods, particularly showcasing notable advantages in computational efficiency and model complexity. These findings underscore the potential of MAF-Net in the field of action recognition, especially in scenarios with high real-time requirements such as IoT and human-computer interaction, where MAF-Net provides a more efficient and accurate solution.

The three main contributions of the paper can be summarized as follows:

1. Proposed an innovative multimodal fusion framework, MAF-Net: Multimodal fusion framework named MAF-Net, which combines multi-frame RGB images and skeleton sequences, significantly reducing computational complexity while preserving key spatiotemporal information. By leveraging self-attention and cross-attention mechanisms, the framework enhances the correlation between the RGB video modality and the skeleton sequence modality, achieving a well-balanced trade-off between performance and computational efficiency.

2. Introduced a skeleton-guided attention mechanism: We innovatively introduce a skeleton-guided attention mechanism that enables the RGB stream to focus on interaction regions between the human body and objects, compensating for the lack of temporal information in RGB images. By generating a skeleton attention mask based on a Gaussian distribution, the mechanism guides the RGB feature extraction process, effectively capturing crucial details and interaction information in the actions.

3. Extensive experimental validation: We conduct extensive experiments on three public datasets, NTU RGB+D, SYSU, and UTD-MHAD, to validate the effectiveness and superior performance of MAF-Net.

## Related work

### 3D skeleton-based action recognition

In recent years, 3D skeleton-based action recognition has emerged as a prominent research direction due to its robustness against external conditions such as background, lighting, and viewpoint variations. Methods in this domain are primarily categorized into three major approaches: those based on Recurrent Neural Networks [30–32], Convolutional Neural Networks (CNN) [33–35], and Graph Convolutional Networks (GCN) [36–38]. Each of these methods leverages the structural and temporal characteristics of skeletal data to enhance recognition performance, offering distinct advantages in handling the inherent challenges of motion data analysis.

#### RNN-based methods.

Recurrent Neural Network (RNN)-based methods have been widely used in 3D skeleton-based action recognition due to their ability to model temporal sequences. By using recursive connections, RNNs process sequential data effectively, as demonstrated by standard RNNs, Long Short-Term Memory (LSTM) [39], and Gated Recurrent Units (GRUs) [40–42], which address issues such as vanishing gradients and long-term dependency modeling. Despite their effectiveness in capturing temporal dynamics, RNN-based methods often struggle with spatial modeling, limiting their performance compared to other approaches [43–45]. To overcome these limitations, researchers have proposed modifications like two-stream RNN architectures that model both spatial configurations and temporal dynamics simultaneously [46]. Furthermore, attention mechanisms have been integrated into RNNs to enhance spatial-temporal modeling by focusing on informative joints.

In response to challenges in standard RNNs, including gradient explosion and vanishing, new architectures like the Independently Recurrent Neural Network (IndRNN) [47] were developed, which allow for more robust and deeper RNNs, improving the handling of long sequences. Additionally, spatial transformations and attention mechanisms were incorporated to focus on critical joints and mitigate noise in skeleton data [48,49]. These innovations have expanded the applicability of RNNs to complex tasks, although issues with spatial modeling remain, prompting ongoing research into enhancing RNN architectures.

#### CNN-based methods.

CNN-based methods for skeleton-based action recognition typically transform 3D skeleton sequences into pseudo-images, where spatial and temporal information is encoded into 2D formats. This allows convolutional neural networks (CNNs) to extract features from skeleton data similarly to how they process regular images. A key challenge with this approach is capturing both spatial relationships between joints and temporal dynamics effectively. Several researchers have addressed this issue, such as Wang et al. [50], who introduced Joint Trajectory Maps (JTM) to represent joint movements as texture images, and Li et al. [51], who used translation-scale invariant mapping to combine spatial and temporal data more effectively.

However, basic CNN models may struggle to capture complex co-occurrence relationships among joints, as they typically focus on local interactions within a limited convolutional kernel. To mitigate this, Chao et al. [52]. proposed a hierarchical method to progressively aggregate contextual information, enhancing feature learning at different levels. Other enhancements include the use of Temporal CNN (TCN) [53] for modeling spatio-temporal cues and the introduction of multi-stream CNN architectures that improve the representation of skeleton data for action recognition.

#### GCN-based methods.

In GCN-based methods for 3D skeleton-based action recognition, the human skeleton is modeled as a graph where joints represent the nodes, and bones or their temporal connections form the edges [54,55]. The adoption of Graph Convolutional Networks (GCNs) effectively captures spatial-temporal relationships between these joints. The Spatial-Temporal Graph Convolutional Networks (ST-GCN) [56], which significantly impacted this field by constructing a spatial-temporal graph that leverages the natural graph-like structure of the human skeleton. This model encodes both spatial and temporal dependencies between joints, leading to notable advancements in performance on action recognition tasks .

Recent improvements focus on optimizing the GCN-based framework for better spatial-temporal representation. For instance, the Action-Structural Graph Convolutional Network (AS-GCN) introduces multi-task learning to predict future poses, while 2s-AGCN adapts the graph structure during training for dynamic connections between joints [57]. These methods address challenges such as how to effectively represent the skeleton’s inherent graph structure and the need for adaptability to noisy or incomplete data [58]. Despite these advancements, key challenges remain in further optimizing GCNs to fully capture the temporal and spatial nuances of skeleton-based action recognition

### Multimodal action recognition

Recent advances in multimodal fusion have significantly contributed to the field of action recognition [59,60]. By integrating multiple sensory modalities, such as video, RGB images, skeletal data, audio, and text, researchers have substantially improved both the accuracy and robustness of action recognition systems. For instance, Zhou et al. [61] proposed a dance motion detection system based on multimodal fusion, which combines multi-feature video analysis with Computer-Aided Design (CAD) techniques to achieve more precise action recognition. This system enhances the perception of complex actions through the collection and processing of multimodal data. Similarly, Tran et al. [62] developed a unified multimodal consistency framework aimed at addressing human activity recognition in videos. By maintaining consistency across different modalities, this framework significantly improves action localization and group activity recognition, demonstrating high accuracy and robustness in experimental results.

Other studies have also highlighted the broad applicability and potential of multimodal fusion techniques. Rehman et al. [63] proposed an algorithm that integrates RGB imaging, skeletal tracking, and pose estimation, demonstrating significant performance improvements on the UTD multimodal human action dataset. Liu et al. [64] addressed limitations in the multimodal fusion process by introducing an adaptive multimodal graph representation fusion method, which enhances action recognition accuracy by combining skeletal data with motion trajectories. Additionally, He et al. [65] explored the application of multimodal fusion for in-vehicle human action recognition, comparing early and late fusion strategies, and revealed the significant impact of different fusion strategies on overall recognition performance. Future research will continue to investigate optimization of multimodal fusion techniques to tackle the challenges posed by complex scenarios and action recognition tasks.

### Transformer-based methods

Transformer-based methods have gained significant attention in the field of 3D skeleton-based action recognition due to their ability to capture long-range dependencies and global relationships, especially using the multi-head self-attention (MSA) mechanism [66–68]. These methods demonstrate superior performance in processing sequences by aggregating spatial-temporal data through attention-based approaches. Notable architectures, such as the Self-Attention Network (SAN) and the Spatial-Temporal Transformer Network (ST-TR) [69], have introduced novel ways to model the spatial-temporal correlations and dependencies between joints, improving the accuracy of action recognition models. However, the main challenge remains in effectively capturing high-dimensional semantic information and modeling intricate spatial relationships within skeleton data .

Hybrid models combining Transformers with GCNs or CNNs have also been explored to leverage the strengths of each architecture, allowing a more comprehensive framework for 3D skeleton-based tasks [70,71]. Such models benefit from Transformers’ global relational modeling abilities while mitigating their limitations in spatial encoding. For instance, the decoupled spatial-temporal attention network [72] and TemPose [73] focus on improving temporal and spatial decoupling, demonstrating strong potential in handling action sequences of varying lengths. These approaches collectively enhance both computational efficiency and recognition accuracy, showcasing the growing dominance of Transformers in this domain .

## Methods

**Fig 2:**
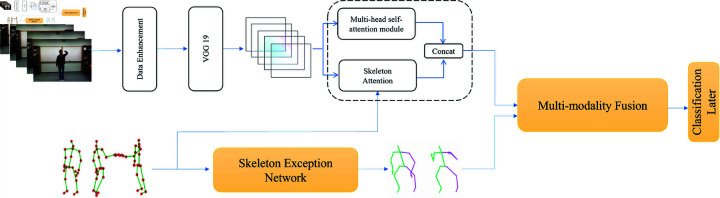
The framework of the MAF-Net.

In this section, we describe the comprehensive approach adopted for data preparation, feature extraction, and model design in our multimodal human activity recognition system, as inlustrated in Fig 2. The proposed method focuses on leveraging both RGB and skeletal data to enhance recognition accuracy and robustness. By incorporating optical flow for key frame extraction, skeletal attention mechanisms, and feature fusion techniques, we aim to address the challenges of capturing temporal and spatial dynamics in complex action sequences. Furthermore, we detail the data augmentation strategies, feature selection processes, and the overall model architecture, including the fusion of RGB and skeletal modalities, to optimize the performance of the recognition task.

### Data preprocess

This section provides a detailed explanation of the key steps involved in preprocessing the RGB data for activity recognition using a sequence modeling approach. Proper data preparation plays a vital role in the overall process, as it is essential for extracting meaningful features from the raw data. These features, when effectively extracted, significantly contribute to the success of the sequence model by improving its ability to recognize patterns and ensure optimal performance during activity recognition tasks.

#### Key frame extraction and uniform sampling based on optical flow.

First, the average motion intensity of each frame is calculated using the optical flow method. Specifically, for each frame and the previous frame, the optical flow vectors u(x,y) and v(x,y) are computed for each pixel, representing the motion components in the x and y directions, respectively. Then, the average motion intensity for the entire frame is calculated using the following formula:


$$\text{Motion Intensity}=\frac{1}{N}\underset{x,y}{\sum }\sqrt{u{\left(x,y\right)}^{2}+v{\left(x,y\right)}^{2}}$$

where N is the total number of pixels in the frame, and $\sqrt{u{\left(x,y\right)}^{2}+v{\left(x,y\right)}^{2}}$ represents the motion intensity of each pixel. Through this process, the motion intensity of each frame can be obtained, indicating the level of activity change in the video for each frame.

Next, based on the calculated motion intensity, all frames are ranked, with frames exhibiting higher motion intensity selected as candidate key frames. These frames represent portions of the video with significant activity changes. To avoid concentrating key frames within specific time intervals, frames are uniformly selected from the sorted candidate frames based on a sampling interval. The sampling interval is computed using the following formula:


$$\text{Sampling Interval}=\frac{\text{Total Number of Frames}}{T}$$

where the Total Number of Frames refers to the total number of frames in the video, and T is the desired number of key frames to be extracted. This sampling interval ensures that the key frames are reasonably distributed across the video timeline, thereby covering the major activity changes throughout the video.

#### Data enhancement.

To enhance the robustness and adaptability of action recognition systems, we propose an improved skeletal data augmentation method, with a focus on optimizing viewpoint expansion and noise handling. For viewpoint expansion, we build upon existing rotation matrix-based augmentation techniques by incorporating additional angular variations, such as $\pm 90^{\circ }$ and 180${}^{\circ }$ rotations, and introducing multi-axis simultaneous rotations, thereby extending the range of perspectives covered. This improvement enables the model to better handle multi-view inputs in complex environments, increasing the accuracy of action recognition. Furthermore, to enhance model stability in real-world applications, we introduce random noise into the skeletal data by applying small perturbations to joint coordinates, simulating sensor errors or jitter during data collection. This approach improves the model’s tolerance to imprecise data and noise, ensuring robust performance under varying noise conditions. The combination of viewpoint expansion and random noise significantly increases the diversity and complexity of skeletal data, thereby improving the system’s recognition performance under multi-view and noisy conditions, and enhancing its robustness and generalization capabilities in complex real-world scenarios.

In the selected RGB frames, since the human body occupies only a small portion of the image, we propose a projection-based cropping method for image preprocessing. This method utilizes skeleton data to define the bounding box of the human body, and subsequently crops the image based on this bounding box. Assuming the bounding box has a width of *w* and a height of *h*, the image is cropped into a region with dimensions of $w+w^{\prime }$ and $h+h^{\prime }$, where $w^{\prime }$ and $h^{\prime }$ are random values between 100 and 300 pixels, effectively augmenting the dataset. Specifically, 3D skeleton coordinates are projected onto RGB frames using camera parameters to calculate the 2D pixel coordinates of the human body, which are then used to determine the cropping region. Unlike traditional random cropping centered on the image, this method focuses on the human body, aligning the skeleton data with image coordinates, thereby forming part of the action recognition pipeline. Its advantage lies in ensuring that the human body occupies the main part of the cropped image, reducing background noise and facilitating the application of skeleton attention mechanisms. However, applying projection cropping and coordinate alignment for each frame increases computational complexity and may affect the integrity of video representation.

#### 2D pose estimation.

In order to obtain the 2D pose coordinates for each sampled frame, we employ the use of OpenPose, which is a pose estimation algorithm that utilizes deep learning models that have been trained on large-scale datasets comprising labeled human poses. The model processes each frame and identifies the keypoints corresponding to a number of body joints, including the nose, elbows, knees, and shoulders. OpenPose specifically extracts 18 keypoints in the COCO format, which include keypoints like the eyes, ears, wrists, hips, and ankles. These keypoints represent the coordinates of important joints and are used to construct the skeletal structure of the human body in 2D space, as shown in Table 1.

**Table 1:** Keypoint names and their indices.

Keypoint name	Index
Nose	0
Neck	1
RShoulder	2
RElbow	3
RWrist	4
LShoulder	5
LElbow	6
LWrist	7
RHip	8
RKnee	9
RAnkle	10
LHip	11
LKnee	12
LAnkle	13
REye	14
LEye	15
REar	16
LEar	17

### Data structure and features

Once all T frames of 2D pose coordinates are obtained, the next step involves extracting features relevant to activity recognition, as shown in Table 2. This process includes computing the normalized Euclidean distances, improving angle-based features, and introducing velocity and acceleration features to comprehensively describe human motion behavior.

**Table 2:** Structure and characteristics of the data.

Component	Description	Number of features
Frame identifier	A sequential integer (starting from 1) representing the position of the frame in the video.	1
2D keypoint coordinates	A set of 18 keypoint positions, each represented as a pair of (x, y) coordinates, identifying various body joints.	K = 18 (x, y values)
Joint angles	A set of angles computed between pairs of keypoints using trigonometric methods such as cosine similarity.	$K\;\times \;\frac{\left(K-1\right)}{2}=18\;\times \;\frac{\left(18-1\right)}{2}=153$
Scaled distances	A set of Euclidean distances between keypoints, normalized by a reference distance for consistency across frames.	$K\;\times \;\frac{\left(K-1\right)}{2}=18\;\times \;\frac{\left(18-1\right)}{2}=153$
Change in velocity	A series of acceleration values, derived from the difference in velocity between consecutive frames.	K = 18
Total number of features	The cumulative count of all keypoint coordinates, joint angles, distances, and accelerations.	343

In order to mitigate the scale variations caused by factors such as individual height or camera distance, the 2D coordinates of body parts are first normalized. Specific anatomical landmarks, such as the distance between the left and right shoulders, can be chosen as the normalization factor. Let the coordinates of key points A and B be $\left(A_{x},A_{y}\right)$ and $\left(B_{x},B_{y}\right)$, and the Euclidean distance between these two points is calculated as:


$$\text{distance}(A,B)=\frac{\sqrt{{\left(B_{x}-A_{x}\right)}^{2}+{\left(B_{y}-A_{y}\right)}^{2}}}{d_{\text{ref}}}$$

where $d_{\text{ref}}$ is a reference distance, such as the distance between the shoulders or the hips. This normalization factor reduces the influence of varying body sizes, making the distance features more consistent across individuals.

#### Relative angles.

The calculation of relative angles can be enhanced using the cosine law. Compared to the simple arctangent function, the cosine law provides a more accurate calculation of the angle between two vectors. For two key points A and B with coordinates $\left(A_{x},A_{y}\right)$ and $\left(B_{x},B_{y}\right)$, we can treat these points as a vector, and the angle between this vector and a reference vector (e.g., the vertical or horizontal axis) can be calculated using the following formula:


$$\cos(\theta )=\frac{A\cdot B}{∥A∥∥B∥}=\frac{\left(B_{x}-A_{x})(R_{x}-A_{x})+(B_{y}-A_{y})(R_{y}-A_{y}\right)}{\sqrt{{\left(B_{x}-A_{x}\right)}^{2}+{\left(B_{y}-A_{y}\right)}^{2}}\;\times \;\sqrt{{\left(R_{x}-A_{x}\right)}^{2}+{\left(R_{y}-A_{y}\right)}^{2}}}$$

where $R_{x}$ and $R_{y}$ are the coordinates of the reference vector, and θ is the angle between the two vectors. By calculating the angles between key points, relative changes in human posture can be more accurately captured.

#### Speed characteristics.

In addition to static distance and angular features, dynamic features are also crucial. For each key point, velocity features can be computed based on positional changes between consecutive frames. Let the coordinates of key point A at frame t and frame t+1 be $\left(A_{x}^{t},A_{y}^{t}\right)$ and $\left(A_{x}^{t+1},A_{y}^{t+1}\right)$, respectively, then the velocity can be calculated using the following formula:


$$\text{Velocity}=\frac{\sqrt{{\left(A_{x}^{t+1}-A_{x}^{t}\right)}^{2}+{\left(A_{y}^{t+1}-A_{y}^{t}\right)}^{2}}}{\Delta t}$$

where Δt represents the time interval between consecutive frames. By incorporating velocity features, variations in the speed of human movements, such as running or jumping, can be effectively captured.

### Optimal feature determination

**Fig 3:**
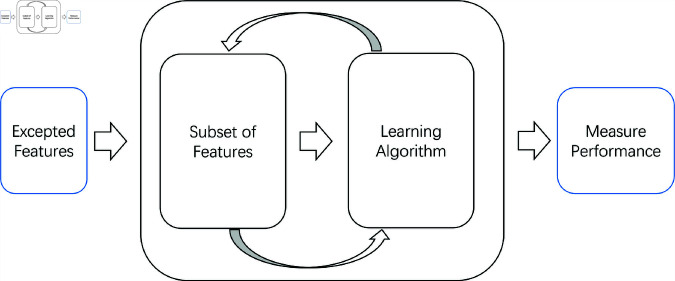
Feature selection technique.

In order to improve the efficiency and effectiveness of the activity recognition model, we implement a feature selection approach. that combines L1 regularization (Lasso) with Recursive Feature Elimination (RFE), the process is shown in the Fig 3. L1 regularization introduces sparsity constraints, automatically shrinking the weights of less important features to zero, thereby effectively reducing the dimensionality of the feature set. This regularization technique preserves the most critical features for model prediction and minimizes redundant information. Subsequently, Recursive Feature Elimination (RFE) is employed to further remove features with minimal contribution to the model’s prediction, ensuring that only the most discriminative subset of features is retained.

In our activity recognition task, each frame contains 283 features, including frame index, angles, and distances between body parts. Although these features comprehensively represent the temporal variations in human posture, not all features are equally important for recognizing specific activities. A combination of Lasso and Recursive Feature Elimination (RFE) can efficiently select the most relevant features for activity recognition, thereby optimizing model performance and enhancing interpretability. This approach first reduces the feature dimensionality using Lasso, followed by a more refined selection of the remaining features through RFE. Subsequently, based on the selected optimal feature subset, a simplified Bidirectional LSTM model is trained to improve the accuracy and efficiency of human activity recognition. While increasing the number of features may extend the feature selection process, this method ensures model consistency during validation and testing phases, maintaining generalization capability without being affected by inconsistent feature sets.

After preprocessing, the dataset is divided using stratified sampling to ensure that the proportion of samples in each class is consistent across the training, validation, and test sets, thus avoiding model bias caused by class imbalance. Specifically, 70% of the data is allocated for training, 15% for validation, and 15% for testing. This allocation allows the model to be trained on representative data while reserving sufficient validation and test data for hyperparameter tuning and performance evaluation. To further enhance the robustness of the model, K-fold cross-validation is applied on the training set, instead of relying on a single fixed validation set. By repeatedly validating the model’s performance within the training set, cross-validation aids in better hyperparameter selection and improves generalization. The test set remains independent throughout, ensuring a fair evaluation of the final model’s performance.

### The RGB frame stream

In our approach, the RGB stream is composed of three primary elements: the foundational convolutional layers, a self-attention mechanism, and a skeletal attention mechanism. We utilize the VGG19 network as the core convolutional layer to derive feature maps. Both the self-attention and skeletal attention modules are structured to produce attention weights, allowing the model to concentrate more effectively on key regions of the image. Compared to other convolutional architectures, the depth and multi-layered design of VGG19 enables it to achieve strong feature extraction, delivering rich feature representations for the subsequent attention processes.

### Multi-head self-attention module

**Fig 4:**
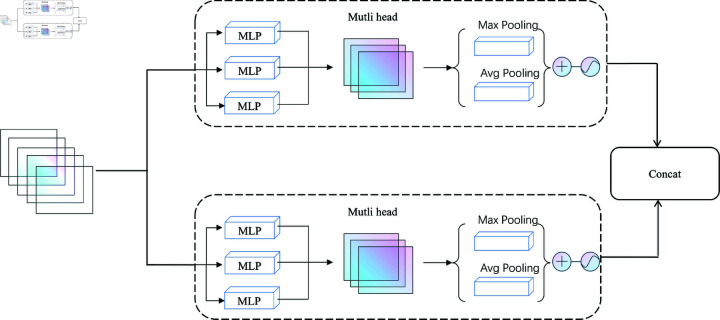
The framework of multi-head self-attention module.

Drawing inspiration from techniques employed in human re-identification tasks for extracting body part features, we introduce a multi-head self-attention mechanism to analyze the feature maps derived from raw RGB frames, as illustrated in Fig 4. The advantage of the multi-head self-attention mechanism lies in its ability to allow the model to focus on multiple regions of the image from different perspectives or scales simultaneously, thus capturing more comprehensive feature representations, particularly enabling effective attention to different parts of complex actions.

Specifically, the input feature map $F_{\text{in}}\in {\mathbb{R}}^{C\;\times \;W\;\times \;H}$ (where C represents the number of channels, and W and H denote the width and height of the feature map) is first transformed through linear projections to generate the Query, Key, and Value matrices. The formulation for these matrices is as follows:


$$Q^{k}=W_{q}^{k}F_{\text{in}},\;K^{k}=W_{k}^{k}F_{\text{in}},\;V^{k}=W_{v}^{k}F_{\text{in}}$$

where $W_{q}^{k}$, $W_{k}^{k}$, and $W_{v}^{k}$ are the learned weights for each attention head. These matrices allow the model to compute correlations between features across different spatial locations. The attention score for each head is computed using the scaled dot-product attention mechanism:


$${\text{Attention}}^{k}(Q^{k},K^{k},V^{k})=\text{Softmax}\left(\frac{Q^{k}{\left(K^{k}\right)}^{T}}{\sqrt{d_{k}}}\right)V^{k}$$

where $d_{k}$ represents the dimensionality of each head, and the scaling factor $\sqrt{d_{k}}$ controls the impact of increased dimensionality. The Softmax function calculates attention weights for each spatial position, reflecting the network’s focus on different parts of the human body.

Multi-head attention is performed in parallel across multiple heads, each focusing on different feature regions. The outputs from all attention heads are concatenated and projected back into the original feature space through a linear transformation:


$$\text{MultiHead}(F_{\text{in}})=W_{o}[{\text{Attention}}^{1},{\text{Attention}}^{2},\ldots ,{\text{Attention}}^{h}]$$

To further emphasize human body features and suppress background information, a 1×1 convolutional layer is introduced to generate a self-attention mask $M_{\text{self}}$, constrained between [0, 1] using the Sigmoid activation function:


$$M_{\text{self}}=\sigma ({\text{Conv}}_{1\;\times \;1}(F_{\text{in}}))$$

Finally, the self-attention mask $M_{\text{self}}$ is applied to the original feature map $F_{\text{in}}$ through element-wise multiplication to produce the final feature map $F_{\text{RGB\_self}}$:


$$F_{\text{RGB\_self}}=M_{\text{self}}⊙F_{\text{in}}$$

This multi-head self-attention mechanism enables the network to extract features from different body parts and spatial regions simultaneously, significantly improving the recognition accuracy of complex human actions.

### Skeletal attention module

**Fig 5:**
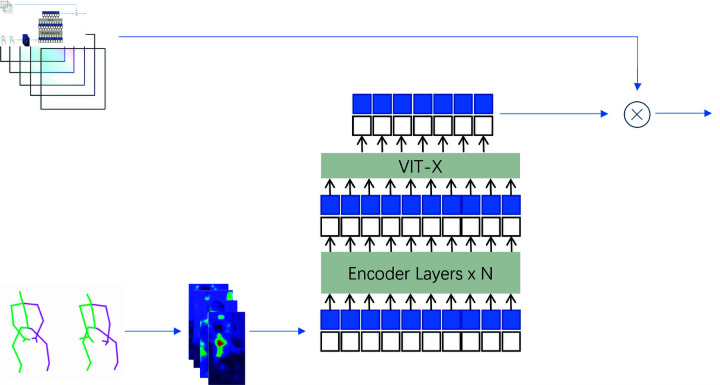
The framework of multi-head self-attention module.

We designed a skeleton attention module that integrates single-frame RGB images with skeleton sequences as a critical step in early feature fusion, as illustrated in Fig 5. Since static RGB frames lack temporal information, the skeleton sequence effectively guides the model to focus on the regions of interaction between the human and objects, complementing the temporal aspect. By computing the movement distance of joints, the module identifies the most salient parts of the activity and generates a skeleton attention mask, assigning higher weights to these regions, thus aiding the RGB stream in capturing complex motion features more effectively.

we fuse single-frame RGB images with skeleton sequences as part of the early feature fusion stage. Since static RGB frames lack temporal information, skeleton sequences are utilized to guide the image in focusing on human-object interaction areas, thus complementing temporal information. First, the joint with the largest movement $j_{\text{max}}$ in the skeleton is computed, as given by the following equation:


dmax= ∥J1,jmax−Jmiddle,jmax∥2jmax= arg ⁡ max ⁡ j ∥J1,j−Jmiddle,j∥2

where $J_{1}$ and $J_{\text{middle}}$ denote the 3D joint coordinates in the skeleton frame and the mid-point of the RGB frame, respectively. By determining the movement of each joint, the joint with the greatest variation $j_{\text{max}}$ is identified.

Next, a skeleton attention mask $M_{\text{ske}}\in {\mathbb{R}}^{1\;\times \;W\;\times \;H}$ is generated. During the generation of the skeleton attention mask, a Gaussian function is applied to smooth the region around the joint with the largest movement, ensuring that the attention weights are more continuous:


$$M_{\text{ske}}^{p}=\exp\left(-\frac{∥p-j_{\text{max}}{∥}^{2}}{2\sigma^{2}}\right)$$

where p represents the pixel location in the mask, and σ controls the spread of the Gaussian distribution. This approach ensures that regions near the joint with the largest movement receive higher weights, while the weights smoothly decrease spatially, preventing neglect of other important regions. The generated skeleton attention mask is then resized to match the spatial dimensions of the feature map, producing the final skeleton attention weights.

Finally, the skeleton-related features $F_{\text{ske}}\in {\mathbb{R}}^{C\;\times \;W\;\times \;H}$ are obtained by element-wise multiplication of the skeleton attention weights and the input feature map $F_{\text{in}}$:


$$F_{\text{RGB\_ske}}=M_{\text{ske}}⊙F_{\text{in}}$$

Guided by the skeleton attention map, the RGB stream is able to more effectively capture human-object interaction features, improving the model’s comprehension of intricate actions.

### Post-fusion module

For action classification, using skeleton data to drive RGB attention, the characteristics of the two subnetwork streams are integrated. Since conventional decision-making fusion methods are highly dependent on specific data sets, we present a feature fusion method inspired by multi-stream fusion approaches to effectively utilize the complementary information provided by the two modes. Both LSTM-based and GCN-based fusion methods are implemented.

#### LSTM—based on fusion module.

Since the skeletal flow features are condensed into a single channel dimension $C_{S}$, we utilize a post-fusion approach to merge the RGB features $F_{\text{RGB}}$. The RGB features are produced by combining the self-attention features $F_{\text{RGB\_self}}$ with the skeleton-attention features $F_{\text{RGB\_ske}}$. These two sets of features are initially processed through MaxPooling layers to obtain features of dimension C, which are subsequently concatenated to form the RGB stream feature $F_{\text{RGB}}\in {\mathbb{R}}^{C_{R}}$.

For the final integration of skeletal and RGB features, both feature sets are combined using a weighted summation rather than concatenation. Following this, a dropout layer is introduced to prevent overfitting by randomly deactivating neurons during training. Afterward, a fully connected layer is applied with the Exponential Linear Unit activation function to model the complex relationships between temporal skeletal features and spatial RGB features. Lastly, the classification task is performed by employing two fully connected layers followed by a sigmoid function for multi-class classification.

#### GCN—based on fusion module.

In order to more effectively investigate the connection and incorporate synergistic information between the two modalities, we developed a fusion module that considers the unique characteristics of the features. The skeletal features $F_{\text{GCN}}\in {\mathbb{R}}^{C_{S}\;\times \;T\;\times \;V}$ contain spatiotemporal information, whereas the RGB features $F_{\text{RGB}}\in {\mathbb{R}}^{C_{R}\;\times \;H\;\times \;W}$ only contain spatial information. In the context of the fusion module, given that the skeleton attention mechanism has already facilitated the extraction of essential features from the RGB frame stream, our focus is on cross-modal spatial relationships.

In order to derive the RGB feature, two attention maps are averaged to derive the RGB feature $F_{\text{RGB}}\in {\mathbb{R}}^{C_{R}\;\times \;S}$, where S is the product of H×W. For the skeleton features, max pooling is applied to convert the skeleton feature $F_{\text{GCN}}\in {\mathbb{R}}^{C_{S}\;\times \;V}$, which contains solely spatial information. Subsequently, both the RGB and skeleton features are transformed into vectors $f_{\text{RGB}}\in {\mathbb{R}}^{C_{R}}$ and $f_{\text{GCN}}\in {\mathbb{R}}^{C_{S}}$ through global max pooling.

Subsequently, the two feature sets are merged through element-wise concatenation. In the case of RGB features, the skeleton vector, designated as $f_{\text{GCN}}$, is combined with each channel of the RGB features, whereas the RGB vector, identified as $f_{\text{RGB}}$, is merged with each channel of the skeleton features. As a result, the dimensionality of the channels in both feature sets is aligned.

Since the merged features incorporate both spatial information from skeleton flow and RGB flow, two 1×1 convolutional layers Conv are utilized to explore cross-spatial relationships, generating a relational mask $M_{\text{rel}}$:


$$M_{\text{rel}}=\sigma \left(\text{Conv}(F_{\text{com}})\;\times \;\text{Conv}(F_{\text{com}}^{T})\right),$$

where σ denotes the softmax function. The resulting features are then obtained through the following equation:


$$F_{\text{rel}}=M_{\text{rel}}⊙F_{\text{com}},$$

where ⊙ represents element-wise multiplication. Subsequently, the relational feature map is passed through a global average pooling (GAP) layer and two fully connected layers, with final classification performed via a softmax layer. This process yields the final output of the network.

### Train steps

Multimodal fusion module would be removed during initial training phase by omitting the feature fusion component of the skeletal flow and RGB flow, allowing the two sub-networks (skeletal flow and RGB flow) to operate independently. A FC layer and a softmax layer are added to the top of each sub-network.

For sub-network training, a simple early-stage fusion mechanism is designed during the training of the skeletal flow and RGB flow sub-networks, allowing partial interaction between the RGB and skeletal flows in the early stages of training to lay a better foundation for later fusion. The weights of each network are saved, but the weights of the FC and softmax layers are not.

The next phase involves gradually unfreezing sub-network weights and training the fusion module. After independently training the sub-networks and obtaining their initial weights, the training of the multimodal fusion module begins. To enhance the alignment between the sub-network and fusion module features, we progressively unfreeze some of the sub-network weights (e.g., the convolutional layers or feature extraction layers close to the top layer) during the training of the fusion module, allowing the sub-networks to further adjust according to the fused features. This approach allows the sub-networks to dynamically adapt to the changes in the fusion module, making the multimodal fusion process more seamless and improving the overall model’s performance and feature alignment accuracy.

Finally, the entire network, including the sub-networks and the multimodal fusion module, undergoes fine-tuning to ensure that the final fused features and sub-network features work in better coordination.

## Experiments

### Dataset description

The NTU RGB+D dataset is another important benchmark in the human action recognition field, containing 60 action classes with a total of 56,880 video samples. This dataset includes various modalities such as skeleton, depth, infrared (IR), and RGB video. Fifty action classes are performed by a single subject, while ten involve two-person interactions. Each action includes data from 25 joints, providing rich spatiotemporal information. The dataset provides two standard evaluation protocols: Cross-Subject, which divides 40 subjects into training and testing groups, and Cross-View, which uses data from cameras 2 and 3 for training and data from camera 1 for testing. The multimodal nature of the NTU RGB+D dataset makes it a challenging and valuable benchmark for action recognition research.

The UTD Multimodal Human Action Dataset (UTD-MHAD) is a publicly available dataset designed specifically for human action recognition research, covering multimodal data collection. It consists of 8 subjects (4 male and 4 female) performing 27 different actions, with each subject repeating each action 4 times, yielding 861 valid samples (excluding 3 damaged ones). The UTD-MHAD dataset includes four synchronized data modalities: RGB video, depth video, skeleton data, and signals from inertial sensors. RGB and depth video were captured using a Microsoft Kinect camera, while inertial sensors were worn on the subject’s right wrist or thigh, depending on the action. The Kinect camera was placed approximately 3 meters away from the subject to capture full-body movements. The dataset was recorded at a frame rate of 30 frames per second with a resolution of 640  ×  480 pixels, making it particularly suitable for applications involving sensor fusion and human action recognition.

The SYSU dataset focuses on human-object interaction action recognition, comprising 12 actions performed by 40 subjects, with each action involving 20 joint points. The dataset contains 480 action sequences, all related to interactions with six objects: phone, chair, backpack, wallet, cup, broom, and mop. The SYSU dataset adopts two standard evaluation protocols: Setting-1, which randomly splits the action sequences into training and testing sets, and Setting-2, which randomly assigns subjects into training and testing groups. In both settings, 30-fold cross-validation is conducted to ensure the stability and reliability of the evaluation results. This dataset is particularly well-suited for tasks related to action recognition involving human-object interactions.

The MMAct dataset is a multi-modal activity recognition dataset containing 37 daily life activities, categorized into three groups: 16 complex activities (e.g., carrying), 12 simple activities (e.g., kicking), and 9 desk activities (e.g., using a computer). The dataset consists of 37,000 video clips from 20 subjects, with each activity performed five times by each subject. It is recorded using seven modalities, including RGB videos from four viewpoints, acceleration, gyroscope, and orientation data. The RGB videos are captured at a resolution of 1920x1080 pixels with a frame rate of 30 frames per second. The inertial sensor data is collected from a smartphone placed in the subject’s pocket and a smartwatch, which record acceleration, gyroscope, and orientation data, resulting in data from a total of four sensors. To increase the diversity of scenes and viewpoints, the dataset includes four different scenes, each with four camera perspectives, providing a rich variation for assessing the robustness of the model. Some of the data suffers from visual occlusions, offering a challenge for activity recognition with incomplete or occluded data. In the experiments, we follow the cross-subject and cross-view split protocol from the original paper. Additionally, since the dataset lacks skeleton sequences, we generate skeleton data from the RGB videos using OpenPose, providing complete input for subsequent analysis.

### Implementation details

The model is implemented based on PyTorch and trained using two Nvidia GTX 4090 GPUs. To optimize training performance, we utilize the AdamW optimizer with an initial learning rate of $5\;\times \;10^{-5}$, and reduce the learning rate by a factor of 0.5 every 15 epochs to ensure smooth convergence and increased training stability. To enhance the robustness of the model, we refined the coordinate transformation strategy and improved the VA-pre preprocessing method. By setting the origin of the coordinate system to the midpoint of each action sequence, the model’s adaptability to variations in viewpoints and camera positions is strengthened. In subsequent frames, the skeleton coordinates are dynamically adjusted based on their relative positions, thus better capturing the relative displacement and spatiotemporal features of the actions. These improvements result in a more precise and robust performance of the model when handling multimodal action recognition tasks.

## Experimental results

### Comparison against other methods

#### Results on NTU RGB+D.

We compared the accuracy on the NTU RGB+D dataset for Cross-Subject and Cross-View settings, encompassing several state-of-the-art (SOTA) methods, as shown in Table 3. The experimental results demonstrate that GCN-based models outperform RNN-based models in regional relationship reasoning, leading to superior performance in action recognition tasks. For instance, MAF-Net improves accuracy by 4.7% in Cross-Subject evaluation compared to EleAtt-GRU , with only an 8.6G increase in FLOPs.

**Table 3:** Analysis in relation to the most effective methods for NTU RGB+D.

Methods	Accuracy	Parameters	FLOPs
	**Cross-subject / Cross-view**		
VA-LSTM	80.4% / 86.6%	—	—
D-Pose Traversal Conv	77.8% / 83.9%	—	—
DPRL+GCNN	84.5% / 88.8%	—	—
ST-GCN	82.5% / 87.3%	4.1M	17.2G
EleAtt-GRU	81.7% / 87.4%	1.3M	7.7G
DGNN	90.9% / 95.1%	—	—
MS-G3D Net	92.5% / 97.2%	4.2M	49.8G
SGN	90.0% / 93.5%	2.2M	1.8G
Inflated Resnet50	87.6% / 94.2%	47.8M	169G
2D/3D Multitask	86.5% / —	13.1M	108.9G
2 Stream RNN/CNN	84.7% / 94.7%	81.2M	39.6G
Deep-Bilinear	84.0% / 88.1%	—	—
Posemap	92.7% / 96.2%	—	—
MFAS	91.0% / —	52.2M	220.8G
SGM-Net	89.8% / 96.7%	72.6M	170.2G
MMTM	92.9% / —	50.4M	216.5G
JOLO-GCN	91.4% / 96.8%	7.2M	21.2G
MAF-Net w/o LSTM	86.4% / 92.6%	28.4M	16.3G
MAF-Net w/o GCN	90.6% / 97.3%	30.1M	25.5G

To ensure fairness, we primarily compared methods using the same skeleton-stream backbone. For example, when comparing the Bi-LSTM-based 2 Stream RNN/CNN with MAF-Net (also employing Bi-LSTM), MAF-Net demonstrated competitive results. Additionally, compared to SGM-Net (using ST-GCN as the backbone), MAF-Net (also using ST-GCN) showed superior performance across both evaluation metrics while requiring fewer parameters and FLOPs.

These performance results indicate that although SGM-Net leverages the full RGB video modality, MAF-Net, using only single-frame inputs with two-stage feature fusion, surpasses SGM-Net in both performance and computational cost. Moreover, compared to recent fusion methods such as MMTM and MFAS , MAF-Net achieves superior results, with FLOPs being only 11.4% of MMTM and 11.1% of MFAS.

For ST-GCN-based Posemap and JOLO-GCN, the parameters and FLOPs for Posemap were not provided in the literature. Since Posemap utilizes CNNs to extract features from RGB videos, the computational burden is relatively high. JOLO-GCN, which constructs optical flow motion maps around joints and employs a lightweight GCN for feature extraction, has fewer parameters and FLOPs in comparison to MAF-Net. However, the model does not consider the computational expense associated with generating optical flow from images and the joint-guided flow maps.

#### Results on SYSU.

Table 4 presents a comprehensive comparison of MAF-Net against several state-of-the-art methods on the SYSU dataset, detailing the accuracy achieved by each method under two different settings, along with their respective parameter counts and FLOPs. The VA-LSTM and DPRL+GCNN models attained accuracies of 77.9% and 76.5%, while Local+LGN recorded an accuracy of 84.1%. EleAtt-GRU demonstrated strong performance, achieving an accuracy of 86.7% across both settings, with a computational cost of 1.3 million parameters and 7.7 billion FLOPs. LSGM+GTSC achieved an accuracy of 86.8%, while SGN reported accuracies of 82.6% and 84.0%, requiring 2.2 million parameters and 1.8 billion FLOPs. Other notable methods include MTDA and JOULE, which attained accuracies of 80.2% / 85.5% and 80.6% / 85.9%, respectively, but with significantly higher computational demands of 47.6M and 48.4M parameters, and 203.9G and 204.8G FLOPs. MAF-Net configurations also demonstrated competitive performance, with the model without LSTM achieving 81.9% / 83.6% accuracy, using 28.4 million parameters and 16.3 billion FLOPs. In contrast, the version without GCN showed improved accuracy of 86.3% / 88.1%, with 30.1 million parameters and 25.5 billion FLOPs. This analysis underscores the effectiveness of MAF-Net, particularly its GCN component, in achieving high accuracy while maintaining a manageable computational footprint compared to other methods.

**Table 4:** Analysis in relation to the most effective methods for SYSU.

Methods	Accuracy	Parameters	FLOPs
	**Setting-1 / Setting-2**		
VA-LSTM	77.9% / 76.5%	—	—
DPRL+GCNN	77.9% /—	—	—
Local+LGN	84.1% / —	—	—
EleAtt-GRU	86.7% / 86.7%	1.3M	7.7G
LSGM+GTSC	— / 86.8%	—	—
SGN	82.6% / 84.0%	2.2M	1.8G
MTDA	80.2% / 85.5%	47.6M	203.9G
JOULE	80.6% / 85.9%	48.4M	204.8G
Deep-Bilinear	82.5% / 85.2%	—	—
PI3D	— / 86.8%	—	—
MAF-Net w/o LSTM	81.9% / 83.6%	28.4M	16.3G
MAF-Net w/o GCN	86.3% / 88.1%	30.1M	25.5G

#### Results on UTD-MHAD.

The Table 5 presents the performance of various methods on the Cross-Subject task of the UTD-MHAD dataset, comparing skeleton-based (S), RGB-based (R), and multimodal fusion (S+R) approaches. Skeleton-based methods, such as ST-GCN , demonstrate strong performance, with MS-G3D Net achieving an accuracy of 90.5% and SGN reaching 89.0% accuracy. The RGB-based stream, such as Inflated Resnet50, achieves 88.6% accuracy, albeit with a high number of parameters and computational complexity (170G FLOPs). Fusion methods combining skeleton and RGB streams also yield high accuracy, with MFAS and MMTM obtaining 88.0% and 90.9% accuracy, respectively. Furthermore, the table highlights the outstanding performance of the MAF-Net model (Bi-LSTM and ST-GCN), which achieves 91.5% and 92.0% accuracy while maintaining low computational overhead.

**Table 5:** Analysis in relation to the most effective methods for UTD-MHAD.

Methods	Accuracy	Parameters	FLOPs
	**Cross-Subject**		
VA-LSTM	81.4%	—	—
DPRL+GCNN	85.5%	—	—
ST-GCN	83.5%	5.1M	18.2G
EleAtt-GRU	82.7%	2.3M	8.7G
DGNN	85.9%	—	—
MS-G3D Net	90.5%	5.2M	50.8G
SGN	89.0%	3.2M	2.8G
Inflated Resnet50	88.6%	48.6M	170G
2D/3D Multitask	87.5%	14.1M	109.9G
2 Stream RNN/CNN	85.7%	82.2M	40.6G
Deep-Bilinear	85.0%	—	—
Posemap	87.7%	—	—
MFAS	88.0%	53.2M	221.8G
SGM-Net	89.8%	73.6M	171.2G
MMTM	90.9%	51.4M	217.5G
JOLO-GCN	91.4%	8.2M	22.2G
MAF-Net w/ Bi-LSTM (Ours)	91.5%	29.4M	17.3G
MAF-Net w/ ST-GCN (Ours)	92.0%	31.1M	26.5G

#### Results on MMACT.

Table 6 presents the performance of various methods on the MMACT dataset, comparing skeleton-based (S), RGB-based (R), and multimodal fusion (S+R) approaches. Skeleton-based methods, such as JOLO-GCN, achieve remarkable performance, with an accuracy of 91.4%. The RGB-based stream, represented by Inflated Resnet50, achieves an accuracy of 88.6%, though with a significant computational cost (170G FLOPs). Fusion methods that combine skeleton and RGB data also perform well, with MAF-Net w/ Bi-LSTM achieving the highest accuracy of 91.5%, while MAF-Net w/ ST-GCN follows closely with 90.3%. These fusion methods offer a strong balance between high accuracy and computational efficiency, with MAF-Net models maintaining relatively low FLOPs (17.3G for Bi-LSTM and 26.5G for ST-GCN). Overall, the table highlights the competitive performance of skeleton-based, RGB-based, and multimodal approaches, with the MAF-Net models standing out for their superior accuracy and efficient computation.

**Table 6:** Analysis in relation to the most effective methods for MMACT.

Methods	F1-Score(%)	Parameters	FLOPs
TSN	87.2%	—	—
MMAD	81.0%	5.3M	19.3G
EleAtt-GRU	80.5%	2.5M	9.0G
MS-G3D Net	88.8%	5.1M	52.1G
SGN	86.2%	3.5M	3.1G
Inflated Resnet50	89.3%	50.1M	174.9G
2D/3D Multitask	84.5%	15.3M	113.7G
HAMLET	84.2%	80.0M	41.6G
Posemap	86.0%	—	—
MFAS	87.0%	55.0M	230.2G
SGM-Net	89.1%	70.0M	168.3G
MMTM	89.7%	53.0M	215.1G
JOLO-GCN	90.0%	8.5M	23.4G
MAF-Net w/ Bi-LSTM (Ours)	92.0%	30.0M	18.1G
MAF-Net w/ ST-GCN (Ours)	91.1%	32.0M	28.2G

### Ablation study

In the process of key frame extraction from RGB videos, we compared two methods and conducted experiments on the UTD-MHAD, NTU RGB+D, and SYSU datasets. The first method is a single-frame RGB image selection strategy, where for efficiency, we extract only one RGB frame from the entire video. Ablation experiments were performed to evaluate the effect of selecting different frames between 10% and 90% of the video duration on model accuracy. Results showed that frames between 30% and 70% performed similarly, leading us to consistently choose the frame at the 50% position, demonstrating that a single RGB frame contains sufficient spatial information to describe human-object interactions. However, the second method, which involves key frame extraction and uniform sampling based on the optical flow technique, proved to be more advantageous. By calculating the motion intensity of each frame using optical flow, this method effectively identifies the segments of the video with significant motion changes. Frames are then ranked based on motion intensity, and key frames are uniformly selected according to a sampling interval, ensuring reasonable distribution of key frames along the time axis. This approach not only captures essential dynamic information in the video but also avoids the concentration of key frames within a specific time interval. Compared to the single-frame extraction strategy, it provides a more comprehensive representation of motion changes in the video. Ultimately, through analysis, we chose the key frame extraction and uniform sampling strategy based on optical flow to achieve more accurate activity recognition.

### Effectiveness of data enhancement

The Table 7 demonstrates the effectiveness of data enhancement methods across different datasets and settings. Specifically, comparing ST-GCN with and without data augmentation reveals a consistent performance improvement across all benchmarks. For instance, in the NTU RGB+D Cross-Subject setting, data augmentation boosts accuracy from 82.5% to 83.3%, and in the Cross-View setting, the improvement is even more pronounced, increasing from 89.3% to 90.6%. Similarly, for the Xception model, employing projection crop as a data enhancement technique raises the accuracy significantly from 50.9% to 66.4% in the NTU RGB+D Cross-Subject setting. The MAF-Net also benefits from data augmentation, with accuracy increasing from 89.2% to 90.6% in the NTU RGB+D Cross-Subject evaluation and from 84.7% to 86.3% in SYSU Setting-1. Across various datasets, including SYSU and UTD-MHAD, data enhancement strategies consistently contribute to higher classification accuracy, highlighting the critical role of augmentation techniques in improving model robustness and generalization.

**Table 7:** Performance comparison of models with and without data enhancement across multiple datasets.

Methods	NTU RGB+D	NTU RGB+D	SYSU	SYSU	UTD-MHAD	MMACT
ST-GCN	82.5%	89.3%	79.6%	80.8%	82.4%	77.9%
ST-GCN w/ Enhancement	83.3%	90.6%	81.4%	82.2%	83.7%	78.5%
Xception	50.9%	51.2%	53.2%	56.4%	51.8%	49.1%
Xception w/ Enhancement	66.4%	71.8%	63.4%	66.7%	64.2%	55.9%
MFAS	91.0%	94.2%	—	—	91.5%	82.4%
MAF-Net w/o Enhancement	89.2%	96.2%	84.7%	86.1%	88.6%	85.2%
MAF-Net	90.6%	97.3%	86.3%	88.1%	91.0%	89.5

### Effectiveness of self-attention mechanisms

In the experiments conducted on the NTU RGB+D and SYSU datasets, the effectiveness of the self-attention mechanism was evaluated using Bi-LSTM and ST-GCN as backbone networks. As shown in Table 6, the inclusion of the self-attention mechanism significantly enhanced the performance of the backbone networks. For instance, on the SYSU dataset, the accuracy of MAF-Net with the self-attention module improved by 1.8% in the Cross-Subject evaluation compared to the Bi-LSTM version without the self-attention module. This indicates that the self-attention mechanism helps the RGB stream to focus more effectively on regions involving human-object interactions, thereby extracting more representative feature information.

Moreover, the skeleton attention mechanism also led to substantial performance improvements. Specifically, MAF-Net with the skeleton attention module outperformed the ST-GCN version without this module, achieving a 3.0% increase in accuracy on the NTU RGB+D dataset in the Cross-Subject evaluation. This suggests that the skeleton attention mechanism not only facilitates better fusion of RGB and skeleton modalities but also aids the model in distinguishing between foreground and background, thus improving overall action recognition performance.

### Effectiveness of the post-fusion module

In our approach, we assessed the final fusion technique utilizing LSTM and ST-GCN backbone networks on the NTU RGB+D, SYSU, and UTD-MHAD datasets. We contrasted the proposed method with both decision-level fusion and summation-based fusion techniques. The experimental results demonstrate that MMFF achieves superior accuracy on all three datasets compared to other methods. For methods based on Bi-LSTM and ST-GCN, the proposed late fusion module consistently outperforms decision fusion in most cases. For instance, our proposed Bi-LSTM late fusion module improved performance by 5.0% over decision fusion in the Cross-View task of the NTU RGB+D dataset and by 3.8% in Setting-1 of the SYSU dataset. However, in the SYSU Setting-1 evaluation, the best performance was achieved by the decision fusion method, which can be seen as an anomaly, as decision fusion showed limited generalization capability on the larger NTU RGB+D dataset. Moreover, MMFF combined with the proposed late fusion module outperforms summation fusion, with a 2.4% improvement in the Cross-Subject task of the NTU RGB+D dataset and a 2.0% improvement in Setting-1 of the SYSU dataset. Similarly, the late fusion method exhibited significant performance advantages on the UTD-MHAD dataset, further validating the effectiveness of the module across different datasets.

### Visualization

**Fig 6:**
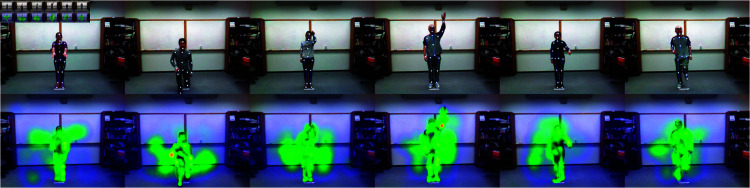
The illustration of the skeleton attention mechanism on UTD-MHAD.

**Fig 7:**
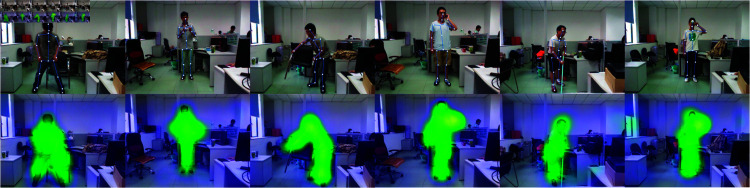
The illustration of the skeleton attention mechanism on SYSU.

**Fig 8:**
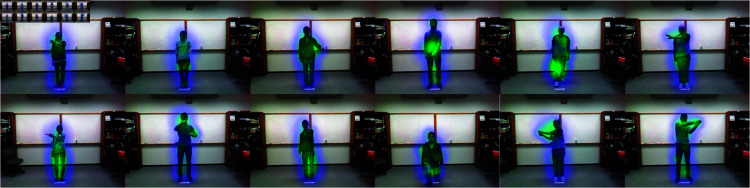
The heatmap of the self-attention on UTD-MHAD.

The visualization results of the self-attention module and the skeletal attention module are shown in Figs 6, 7 and 8, respectively. All images have been cropped according to the implementation details. The results demonstrate that the self-attention module aids the model in better focusing on regions of human-object interaction, while the skeletal attention module effectively guides the model to focus on limb movements, thereby improving the accuracy of action recognition.

## Discussion

The results from our experiments demonstrate the effectiveness of our proposed multimodal fusion approach for human activity recognition, particularly when utilizing skeletal and RGB data streams. By employing advanced techniques such as optical flow-based key frame extraction, enhanced skeletal data augmentation, and feature fusion strategies, our model achieves superior performance across multiple datasets, including NTU RGB+D, SYSU, and UTD-MHAD. These results underscore several critical insights:

- **Key Frame Extraction and Optical Flow**: The motion-based key frame extraction technique using optical flow significantly improves the temporal representation of videos. Compared to single-frame extraction, the optical flow approach ensures that frames with high activity intensity are captured, thereby offering more comprehensive coverage of motion dynamics. This method proved especially beneficial in improving accuracy across larger datasets like NTU RGB+D.
- **Data Augmentation Techniques**: Our findings emphasize the importance of data augmentation, particularly for enhancing the robustness of skeletal and RGB modalities. For the skeletal stream, viewpoint rotation and noise injection improved the system’s adaptability to real-world conditions. Similarly, projection-based cropping on RGB frames, aligned with skeletal data, focused the model’s attention on human-object interactions, reducing the influence of background noise.
- **Self-Attention and Skeletal Attention Modules**: Both attention modules were shown to contribute significantly to model performance. The self-attention module helped the model to focus on critical regions within RGB frames, capturing intricate human-object interactions. The skeletal attention module further aided in aligning key motion areas within the skeletal and RGB streams, leading to improved action recognition accuracy. This was particularly evident in tasks involving fine-grained body movements, where skeletal attention allowed the model to differentiate between similar actions.
- **Fusion Techniques**: The late fusion approach demonstrated clear advantages over decision and summation fusion methods. By effectively integrating features from both the skeletal and RGB streams, this method enabled the model to better utilize complementary information from both modalities, leading to improved performance. The superior results achieved on all datasets, particularly on challenging benchmarks like NTU RGB+D, validate the fusion strategy’s efficacy in multimodal action recognition tasks.
- **Dataset Insights**: The consistent improvement across NTU RGB+D, SYSU, and UTD-MHAD datasets highlights the generalizability of our approach. While the NTU RGB+D dataset, with its complex spatiotemporal data, challenged the model’s ability to capture detailed motion, our fusion methods effectively addressed these challenges. In contrast, the smaller SYSU dataset benefited more from data augmentation, reducing overfitting and enhancing performance in human-object interaction tasks.

### Limitations and future work

Despite the promising results, there are areas for improvement. One limitation of the current approach lies in the computational complexity introduced by the fusion and attention modules, which may hinder real-time applications. Future work could focus on optimizing the computational efficiency of the model, perhaps by introducing more lightweight attention mechanisms or employing knowledge distillation techniques to simplify the model without compromising accuracy.

Additionally, while the optical flow-based key frame extraction showed significant improvement, this method adds preprocessing time, which could be streamlined in future iterations. Exploring alternative motion detection techniques or reducing the need for preprocessing could make the model more practical for real-time applications.

Lastly, integrating more diverse data sources, such as depth information or inertial sensor data, could further improve the robustness of multimodal action recognition systems, particularly in more complex, real-world environments.

## Conclusion

In this work, we presented a comprehensive approach for multimodal human activity recognition using both skeletal and RGB data streams. Our method employs advanced techniques such as optical flow-based key frame extraction, data augmentation, and multimodal fusion with attention mechanisms, resulting in superior performance across three benchmark datasets. The integration of skeletal and RGB features, supported by self-attention and skeletal attention modules, enables the model to capture intricate spatiotemporal relationships and enhance its focus on relevant human-object interactions.

The late fusion strategy used in this work proved to be highly effective, outperforming other fusion techniques and ensuring the complementary strengths of both data streams were utilized to their fullest potential. Our model not only achieves state-of-the-art performance on challenging datasets like NTU RGB+D but also demonstrates strong generalization capabilities across smaller datasets like SYSU and UTD-MHAD.

Looking forward, optimizing the model for real-time performance and extending it to incorporate additional modalities will further enhance its practical applications. Overall, this work provides a robust foundation for future research in multimodal human activity recognition, with promising implications for real-world applications in surveillance, healthcare, and human-computer interaction.
